# Report of a Case of Axillary Aneurism, in the General Hospital, U. S. A., at Picolata, E. F.

**Published:** 1841-09

**Authors:** Charles M’Dougall

**Affiliations:** U. S. Army


### Selections from American and Foreign Journals.

*Report of a Case of Axillary Aneurism, in the General Hospital, U. S. A., at Picolata, E. F.—By Surgeon Charles M'Dougall, U. S. Army.* John Kane, ætat. 24, a private of company K., third artillery, while escorting an officer some weeks since to St. Augustine, was shot by the Indians, and wounded in two places; one ball striking midway between the eighth dorsal vertebra and the angle of its rib, appeared in front, under the integuments, without having penetrated the abdominal cavity; the other, entering about two inches above the lower angle of the scapula, came out anteriorly in a line with, and severing the axillary artery, equidistant from the middle of the clavicle and the axilla. He ran his horse for half a mile, after being shot, when he fell, exhausted from hæmorrhage, and was brought to the general hospital in a state of insensibility. The circulation being extremely weak, skin cold and pallid, a little wine was administered, which, with warm applications to the extremities, soon brought on sufficient reaction to enable the system to recover its lost energies. On making a careful examination of his wounds, a ball was extracted from under the skin of the left side; the integuments around the anterior upper wound, were found enormously distended, having a dark, livid appearance. Life, in this case, has been preserved by the ordinary laws of the natural economy, syncope and pressure from the effused blood. Simple dressings to the wounds, perfect rest, evaporating lotions, moderate pressure, and the usual antiphlogistic means, constituted the treatment.

At the expiration of a fortnight, the effused blood and swelling had disappeared. The external wounds were healthy, and, judging from the small discharge, the internal track of the wounds had united by the first intention. The patient was considered out of all danger, and continued to improve until the end of the fourth week, when a small pulsatory tumor suddenly appeared over the axillary artery, and directly under the external wound. A false aneurism was rapidly forming. The tumor continued to enlarge and pulsate with great violence, although pressure had been made above and upon it, and, after the tenth day, extended above to the clavicle, pushing that bone out of its natural position, downwards, into the axilla, and anteriorly, putting the pectoral muscles and integuments so greatly on the stretch, as to threaten momentarily to give way; pulse at the wrist almost imperceptible; has frequent and violent attacks of neuralgic pains, along the arm, forearm, and fingers; arm insensible to touch, and motionless. Sound, communicated to the ear by the stethescope, like the rushing of fluid at each descent of the piston of a forcing pump. There could be no doubt or hesitancy as to its important character. The locality of the wound—hæmorrhage—distension—absorption of the effused fluid—sudden appearance of a small pulsating tumor—its increase—pulsating equally over the whole circumference—sound—with the entire absence of all the symptoms of suppuration, made it doubly certain that the artery had given way at the wounded point. Apprehending an immediate rupture of the tumor, after consultation with that able officer of the Medical Staff, Assistant Surgeon Hoxton, (who, at the time, was among the number of my patients,) and agreeing that the taking up of the subclavian artery above the clavicle was the only alternative, determined upon the operation on the following day.

Saturday, Dec. 12th.—All the necessary arrangements being made, sixty drops of tinc. opii were given, and as soon as its effects were most apparent, he was laid upon a narrow operating table. After depressing the shoulders as much as possible, and drawing the skin about one-third of an inch over the clavicle, a transverse incision was made directly upon that bone to near its acromial end; this was done, in order to make the first cut with precision. The integuments resuming their natural position, the platysma myoides and superficial fascia were divided by repeated and cautious strokes of the knife to the extent of the first incision. The bleeding from two superficial veins, made it necessary to tie them. The external jugular was carefully avoided. About half an inch of the sterno-cleido-mastoideus was cut, the better to expose the nerves and vessels beneath. After separating the lymphatic glands and fatty tissue from their connexions, the cervical nerves were brought plainly into view, and on the inner side and beneath, to a great depth, was recognized the subclavian artery, which, for the want of a properly curved aneurismal needle, was with some difficulty secured by a ligature.

All pulsation at the wrist and the tumor now ceased. The pain, which continued throughout most of the operation, subsided. The edges of the incision being brought together by three sutures and two adhesive strips, and a light bandage drawn over the whole, the patient was conveyed to bed. ℞ pulv. doveri. grains x. Attendants to watch by the bed side.

13th—Passed a quiet night; no return of the neuralgic pains; no pulse at the wrist; arm still insensible; temperature below natural; some excitement; tongue much furred; bowels have not been opened; V. S. 1 ℔.; ℞. hyd. submur. grs. xv.; ol. Ricini ℥iss, six hours after. Evening—medicine has operated; skin moist; tongue cleaner; no change in the condition of the arm; tumor diminished some; ℞. pulv. doveri, grs. x. at 9 P. M.

14th—Slept well; expresses himself as feeling comfortable; tongue yet furred; ℞. acet. morph., tart., ant. aa grs. ¼, 9. A. M. Electric shocks to the arm. Evening—no apparent change; electric shocks cause an agreeable tingling sensation; looks well; repeat morning's prescription at 9, P. M.

15th—Continues to improve in general appearance; tumor evidently much smaller; feels soft and flabby; ℞. massa. ex. hyd., bi carb. sodæ aa grs. x, at 9, P. M.

16th—Bowels moved; wound examined and dressed. Discharge free and healthy; but a small part of the wound united; a slight blush around the clavicular end of the wound; adhesive strips with light cerated dressings over. Evening—no variation.

17th—Wound dressed; healthy granulations appear; free from excitement; arm has its natural warmth, and fast recovering its sensibility. Tumor daily and rapidly diminishing.

18th—Patient comfortable and cheerful; electric shocks continued; wounds dressed morning and night. 12, P. M.—suddenly called up to visit Kane; found him deluged in blood; breathing laborious; the hæmorrhage had taken place while he was asleep, and was arrested by the syncope; bandage being glued by the drying of the blood, was not disturbed; compress placed over it, with directions to attendants where to apply pressure. The artery had undoubtedly ulcerated at the point where the ligature had been applied.

19th—No bleeding since 12 o'clock last night. Noon—another alarming hæmorrhage, which was arrested by pressure; attendants to keep up constant pressure with the thumb by reliefs; pulse barely perceptible, no bleeding since noon; surface cold. He continued to linger until 12, P. M., when he breathed his last.

*Examination ten hours, post mortem.* An incision eight or nine inches in length was made, extending from the centre of the clavicle, over the top of the tumor, to some distance down the inner part of the arm; the integuments were freely dissected and turned back, the pect. major cut and drawn out of the way. This muscle was attenuated, perhaps by distension and absorption; the blood had been forced through its fibres like through a seive, and in some places incorporated with its substance. The same was the case with the pect. minor. On raising both these muscles, an immense mass of coagulated blood, which appeared partially organized, was disclosed beneath, and contained in an imperfectly formed sac. The sac being washed out and cleaned, brought the parts more fairly into view. The artery was found completely divided, and the ends retracted to the distance of half an inch. Not the slightest adhesion had taken place, the mouths being as patulous as if just cut; veins and nerves perfect, and in position. To expose the parts above the clavicle, an incision was made along the course of the mastoid muscle; commencing about half way between the mastoid process and the sternal extremity of the clavicle, and met by a transverse cut along the whole length of that bone. The fascia, platys. myoides, stern. mastoideus, were divided, and the subjacent parts freely exposed. The artery had ulcerated where the ligature had been applied, presenting a scollopped, uneven edge at both orifices. No trace of adhesive inflammation in it could be detected. The inner coat of the artery had a pale, bleached appearance. A small coagulum of blood rolled out of the orifice nearest the heart, when the finger was pressed upon it. Granulations were found at the bottom of the wound, and had completely filled it, excepting where the ligature was. The surrounding tissues were consolidated by adhesive inflammation, and rendered the dissection tedious and difficult.

The remarkable fact in this case of the perfect want of adhesive inflammation at both points where the artery had been divided, can only be accounted for, on the supposition of some mysterious constitutional influence. Six weeks had elapsed from the reception of the wound. Kane was a large, muscular, healthy man, and in good condition for a surgical operation. The artery was fairly tied with a moderate sized ligature, and with but little disturbance of its natural connexions. Every symptom, subsequent to the operation, appeared favorable to its ultimate success. It is true, that the system had betrayed a certain degree of nervous irritation from the first, distinct from the violent neuralgic pains, and in a greater degree after the operation, but it did not impede the necessary adhesive inflammation in the surrounding parts. The fact of the artery being tied near where the subscapular is given off, is not sufficient to account for the total absence of an effort of nature to unite its sides; inasmuch as it has been satisfactorily proven, that an artery, although tied immediately at the point where a branch is given off, will unite at the compressed part, so as to be secure against all danger. We are therefore constrained to admit that some secret action beyond the reach of medicine or art, exercised a positive influence over the animal economy, inimical to the progress of a healthy cure in all the structures alike.

*January*, 1841.

